# Dormancy, stemness, and therapy resistance: interconnected players in cancer evolution

**DOI:** 10.1007/s10555-023-10092-4

**Published:** 2023-02-09

**Authors:** Federica Francescangeli, Maria Laura De Angelis, Rachele Rossi, Adriano Cuccu, Alessandro Giuliani, Ruggero De Maria, Ann Zeuner

**Affiliations:** 1 Department of Oncology and Molecular Medicine, Istituto Superiore di Sanità, Viale Regina Elena 299, 00161 Rome, Italy; 2 Department of Statistical Sciences, La Sapienza University of Rome, Piazzale Aldo Moro 5, 00185 Rome, Italy; 3 Environment and Health Department, Istituto Superiore di Sanità, Viale Regina Elena 299, 00161 Rome, Italy; 4 Institute of General Pathology, Università Cattolica del Sacro Cuore, Largo Francesco Vito 1, 00168 Rome, Italy; 5 Fondazione Policlinico A. Gemelli IRCCS, 00168 Rome, Italy

**Keywords:** Quiescence, Dormancy, Therapy resistance, Stemness, Cancer stem cells, Tumor relapse

## Abstract

The biological complexity of cancer represents a tremendous clinical challenge, resulting in the frequent failure of current treatment protocols. In the rapidly evolving scenario of a growing tumor, anticancer treatments impose a drastic perturbation not only to cancer cells but also to the tumor microenvironment, killing a portion of the cells and inducing a massive stress response in the survivors. Consequently, treatments can act as a double-edged sword by inducing a temporary response while laying the ground for therapy resistance and subsequent disease progression. Cancer cell dormancy (or quiescence) is a central theme in tumor evolution, being tightly linked to the tumor’s ability to survive cytotoxic challenges, metastasize, and resist immune-mediated attack. Accordingly, quiescent cancer cells (QCCs) have been detected in virtually all the stages of tumor development. In recent years, an increasing number of studies have focused on the characterization of quiescent/therapy resistant cancer cells, unveiling QCCs core transcriptional programs, metabolic plasticity, and mechanisms of immune escape. At the same time, our partial understanding of tumor quiescence reflects the difficulty to identify stable QCCs biomarkers/therapeutic targets and to control cancer dormancy in clinical settings. This review focuses on recent discoveries in the interrelated fields of dormancy, stemness, and therapy resistance, discussing experimental evidences in the frame of a nonlinear dynamics approach, and exploring the possibility that tumor quiescence may represent not only a peril but also a potential therapeutic resource.

## Introduction

Cancer evolution from early- to late-stage disease proceeds with a parallel increase in plasticity and heterogeneity, creating a dynamic network continuously shaped by cell-intrinsic properties and microenvironmental signals [1]. Anticancer therapies crucially influence cancer evolution by inducing dramatic perturbations at the local and systemic level, thus forcing tumor cells to adopt new phenotypes to survive cytotoxic signals. Sequential rounds of chemo/radiotherapy and targeted agents often put the brakes to tumor expansion, extending time to progression and prolonging patient survival. However, cancer cells ultimately develop resistance, and cures for advanced tumors remain uncommon. The generation of therapy resistant cells was classically ascribed to a Darwinian-like selection, similarly to what happens with resistant bacterial strains during antibiotic therapy. However, it is becoming increasingly clear that therapy plays an active (and not simply selective) role in the generation of resistant cells. These cells display a higher expression of stress-response genes, are usually characterized by a quiescent/slow cycling state, and, upon reactivation, give rise to cancers invariantly more aggressive than the initial one [2, 3]. Therefore, the simple Darwinian-like selection paradigm needs to be reconsidered at the light of cancer cell plasticity and heterogeneity [4, 5]. Cancer therapy has been shown to promote both tumor quiescence and stemness, resulting in the survival of slow cycling cancer stem cells (CSCs) with high tumor-repopulating potential [6–9]. Cells with combined properties of therapy resistance, quiescence, and stemness have been identified in many tumors including glioblastoma, melanoma, osteosarcoma, acute and chronic leukemias, and lung, breast, ovarian, colorectal, and pancreatic cancer [6–8, 10–19] (reviewed in [3]). These evidences point to dormancy/quiescence, stemness, and chemoresistance as central and interrelated threads in cancer evolution.

## Quiescence in cancer: an overview

Reservoirs of dormant individuals are present across all the kingdoms of life including viruses, bacteria, fungi, plants, worms, insects, fishes, birds, and mammals [20]. A common feature of dormant forms of life is their increased resilience that allows them to resist major environmental challenges. Usually, dormancy is not only a form of metabolically inactive resilience but also a state endowed with high regenerative potential, as exemplified by plant seeds, fungal spores, or mammalian oocytes. Cancer dormancy occurs at two different levels: the first level, named *tumor mass dormancy*, consists in the whole tumor maintaining an overall constant size due either to stalled growth or to a balance between proliferation and death (for further details see [21]). The second level, called *cancer cell dormancy* or *quiescence* (we will use these terms interchangeably further on), occurs locally and is a process whereby individual tumor cells enter a state of reversible cell cycle arrest [22]. Cancer cell dormancy can occur in the period of latency before the development of a primary tumor and in every stage of cancer evolution ranging from quiescent cells in untreated tumors [12, 23–26] to early metastatic cells [27–29] to drug-tolerant persisters (DTPs) [15] and to disseminated tumor cells (DTCs) [30]. Quiescent/slow cycling cells have been identified even in contexts of rapid disease progression such as advanced (stage IV) tumors [31] and high-grade tumors [32]. Glioblastoma multiforme (GBM) is one of the tumors where quiescent cells have been most thoroughly characterized [33]. In 2012, Parada et al. identified a subset of relatively quiescent GBM CSCs able to sustain long-term tumor growth and to repopulate the tumor upon temozolomide (TMZ) treatment [6]. Ten years later, the same group further analyzed quiescent CSCs isolated from GBM patient-derived xenografts, identifying a 118-gene signature containing several stem cell-associated transcription factors and a specific enrichment of the F3 receptor (coagulation factor III, CD142, tissue factor) [26]. F3^+^ GBM cells survived TMZ treatment and, upon therapy cessation, fueled tumor regrowth, revealing their CSCs nature. In lung cancer, quiescent cells were first identified in patient-derived spheroids and shown to be resistant to chemotherapy resistant but sensitive to Bcl-xL targeting [16]. More recently, Maynard et al. identified quiescent/slow cycling cells expressing an alveolar-regenerative stem cell signature and that were enriched in lung tumors treated with tyrosine kinase inhibitors (TKIs) [34]. In breast cancer, quiescent stem cells were first isolated and characterized by Pece and coworkers as label-retaining cells with a transcriptional profile similar to that of normal quiescent mammary stem cells [14]. Subsequently, quiescent breast CSCs were the object of intense investigations revealing multiple roles in chemoresistance and metastatic dissemination [35]. Altogether, these bodies of evidence support the hypothesis that dormancy is a protective state adopted by tumor cells upon many unrelated treatments, which push stressed cells into prototypical stemness-related gene expression patterns [36].

## A molecular portrait of quiescent cancer cells

Cancer cell dormancy can be defined as a reversible non-proliferative state characterized by enhanced properties of stemness and resilience. Dormancy/quiescence is usually a transient state, although in some cases (such as in tumor latency or in metastatic latency) it can endure for decades or even for the patient’s lifetime. Being a rapidly reversible state, quiescence is determined by a complex set of epigenetic (rather than genetic) modifications that in the last years have been the object of intense investigations. Molecular patterns associated with cancer cell quiescence include the modulation of protein kinases activity, an altered expression of adhesion molecules, anti-apoptotic, and autophagy factors, and the implementation of programs of stemness, epithelial-mesenchymal transition (EMT), and pluripotency [30, 37, 38]. Further mechanisms include alterations in DNA methylation, DNA oxidation, and histone modification [39, 40]. The molecular mechanisms linked to cancer cell quiescence are summarized in Table 1. In addition to cell-intrinsic programs, microenvironmental signals are crucial for the acquisition of dormancy, as discussed in Section 5. EMT is likely the most important gene expression program associated to cellular quiescence [41]. SNAI1, SNAI2, ZEB1, ZEB2, and TWIST1 are the main transcription factors that orchestrate EMT and repress epithelial programs [42, 43]. Among these, ZEB2 has been shown to characterize the transcriptional landscape of quiescent cancer cells (QCCs) in colorectal and lung cancer, thus substantiating its key role in the regulation of cancer cell quiescence [12, 44]. The key role of EMT in regulating cancer cell dormancy has been recently demonstrated by Aouad et al. by using intraductal breast cancer xenografts. Disseminated estrogen receptor-positive (ER^+^) breast cancer cells displayed a slow-growing/quiescent phenotype as compared to triple-negative breast cancer (TNBC) cells. ER^+^ cells had increased ZEB1 and ZEB2 expression and exhibited an EMT phenotype that was reversed by forced E-cadherin expression [45]. Interestingly, prototypical molecular patterns associated to cancer cell dormancy have been found in multiple unrelated tumors [44, 46]. In line with this observation, our recent work identified a common quiescence molecular signature in QCCs isolated from CSC-derived xenografts of lung cancer and colorectal cancer [44]. Such quiescence-associated signature included a core of 688 genes shared between lung and colorectal tumors that emerged with remarkable consistency from QCC gene expression profiles. Genes with an increased expression in QCCs encoded for factors involved in stemness/pluripotency (particularly Krüppel-like factor 4/KLF4, one of the four original reprogramming factors used to generate induced pluripotent stem cells, iPSCs) [47, 48]), TGFβ signaling, EMT, cell adhesion, and chemotaxis. Interestingly, the shared signature of colorectal and lung QCCs included a highly interconnected set of genes involved in embryonic morphogenesis [44]. This finding is in line with recent studies showing that quiescent cells adopt a transcriptional program recalling that of embryonic diapause, a state of suspended development adopted by embryos in response to adverse environmental conditions [49, 50]. Despite significant advances made in the molecular mechanisms of quiescence, a specific marker that can be used routinely to identify the existence of QCCs is still missing. NR2F1 has been proposed as a marker to identify dormant DTCs in the bone marrow of breast cancer patients [51], but it was recently shown to be expressed also in cancer-associated fibroblasts in primary tumors [52]. In colorectal cancer, we have consistently observed QCCs with a ZEB2^+^/Ki67^−^ phenotype in untreated and chemotherapy-treated xenografts. Moreover, patient tumors of the consensus molecular subtype4 (CMS4) display high ZEB2 and low Ki67 expression along with EMT-related and therapy-resistant features [12]. Future studies will be instrumental to identify novel quiescence-specific factors with potential utility both as prognostic markers and as therapeutic targets.

**Table 1:** Molecular pathways and factors involved in cancer cell dormancy

Molecular pathways and proteins involved in dormancy	Tumor type	Representative references
↑p38 MAPK, ↓ERK1/2	Head and neck carcinoma, breast cancer, prostate cancer; melanoma; fibrosarcoma	[53–55]
uPAR/FAK/Src	Hepatocellular carcinoma	[56]
cYES1, YAP	Colon cancer, lung cancer	[57, 58]
SPARC	Prostate cancer	[59]
TGFβ1, TGFβ2	Prostate cancer, head and neck squamous cell carcinoma	[60–62]
NR2F1	Head and neck squamous cell carcinoma, prostate cancer, breast cancer	[51, 63–65]
BMP7, BMP4	Prostate cancer, breast and lung cancer	[66, 67]
CXCL12	Breast cancer, salivary adenoid cystic carcinoma, leukemia	[68–70]
Thrombospondin	Breast cancer	[71]
DIRAS3 (ARHI)	Ovarian cancer, breast cancer, pancreatic cancer, lung cancer	[72–75]
HIF1α	Head and neck squamous cell carcinoma, breast cancer	[23, 24, 76]
Adhesion molecules: vWF, integrins, VCAM	Breast cancer	[77]
GAS6	Prostate cancer	[78, 79]
RAR	Head and neck squamous cell carcinoma, prostate cancer	[64]
LIF	Breast cancer	[80]
WNTs	Lung and breast cancer	[27, 81, 82]
Jagged 1	Leukemia	[83]
ZEB2	Colorectal cancer, lung cancer	[12, 44]
Anti-apoptotic factors: Bcl-xL, p-cRAF, p-ASK1	Colorectal cancer, lung cancer; breast cancer	[12, 16, 49, 58]
Cell cycle inhibitory factors: p27^kip1^, p21^Cip1^, p57^KIP2^, DYRK1A, DREAM complex	Murine leukemia, ovarian cancer, colorectal cancer	[12, 84–87]
Stemness associated factors: Nanog, BMI1, KLF4, AXIN2, LGR5, PLAUR/CD87, CD44, ALCAM/CD166, SOX2, SOX9	Colorectal cancer, lung cancer, head and neck squamous cell carcinoma, breast cancer, glioblastoma, melanoma, colon cancer, ovarian cancer	[3, 6–9, 12, 14, 23–27, 29, 30, 64, 88]
EMT-associated factors: vimentin, SNAI1/2, N-cadherin, TWIST, TG2, ZFP281, ZEB1	Colorectal cancer, ovarian cancer, lung cancer, breast cancer	[12, 41, 45, 58, 62, 89, 90]
Autophagy associated factors: mTOR, beclin1 and VPS34, LKB1-AMPK, IGF2, VEGF, IL8, and IGF1	Colorectal cancer, breast cancer, lung cancer, ovarian cancer, osteosarcoma	[24, 50, 74, 76, 91–93]
DNA methylation, DNA oxidation, histone modifications	Head and neck squamous cell carcinoma, prostate cancer, breast cancer, glioblastoma	[39, 40, 64, 82, 94, 95]
Other epigenetic factors: KDM1B, 2, 3, 5, 6, 7; TET2	Glioblastoma, colorectal cancer, melanoma, lung cancer, breast cancer	[15, 96–104]
lncRNAs and miRNAs	Breast cancer	[105–107]
Embryonic-like programs (↓ cMyc↓ mTOR), morphogenesis pathways	Breast cancer, colorectal cancer, lung cancer,	[44, 49, 50]

The reference list is representative and not comprehensive. We apologize with colleagues whose studies could not be included in the list due to space constraints

Abbreviations: *MAPK*, mitogen-activated protein kinase; *ERK*, extracellular signal-regulated kinase; *uPAR*, urokinase plasminogen activator surface receptor; *FAK*, focal adhesion kinase; Src, Rous sarcoma oncogene; *YES-1*, Yamaguchi sarcoma viral oncogene homolog 1; *YAP*, YES-associated protein; *SPARC*, secreted protein acid rich in cysteine; *TGF*β, tumor growth factor beta; *NR2F1*, nuclear receptor subfamily 2 group F member 1; *BMP*, bone morphogenetic protein; *CXCL*, chemokine (C-X-C motif) ligand; *DIRAS3*, DIRAS family GTPase 3; *ARHI*, aplysiaras homology member I; *HIF*, hypoxia-inducible factor; *vWF*, Von Willebrand factor; *VCAM*, vascular cell adhesion molecule; *GAS6*, growth arrest-specific 6; *RAR*, retinoic acid receptor; *LIF*, leukemia inhibitory factor; *WNTs*, wingless-type MMTV integration site family member 1; *ZEB1 and ZEB2*, zinc finger E-box binding homeobox 1 and 2; *Bcl-xL*, B-cell lymphoma-extralarge; *p-cRAF*, phosphocellular rapidly accelerated fibrosarcoma; *p-ASK*, phospho-apoptosis signal-regulating kinase; *cMyc*, cellular homolog of avian myelocytomatosis viral oncogene; *DYRK*, dual-specificity tyrosine phosphorylation-regulated kinase; *DREAM*, dimerization partner, *RB-like*, E2F and multi-vulval class B; *Nanog*, NANOG homeobox; *BMI1*, B cell-specific Moloney murine leukemia virus integration site 1; *KLF4*, Krϋppel-like factor 4; *AXIN2*, axis inhibition protein 2; LGR5, leucine-rich repeat containing G protein-coupled receptor 5; *PLAUR*, plasminogen activator, urokinase receptor; *ALCAM*, activated leukocyte cell adhesion molecule; *SOX*, transcription factor sex determining region (Y SRY)-box; *SNAI*, snail family transcriptional repressor; *TWIST1*, twist family BHLH transcription factor 1; *Smad*, small mother against decapentaplegic; *PAI1*, plasminogen activator inhibitor-1; *TG2*, transglutaminase 2; *ZFP281*, zinc finger protein 281; *mTOR*, mammalian target of rapamycin; *VPS34*, vacuolar protein sorting 34; *LKB1*, liver kinase B1; *AMPK*, AMP-activated protein kinase; *IGF*, insulin-like growth factor; *VEGF*, vascular endothelial growth factor; IL, interleukin; *KDM*, lysine-specific demethylase; *TET2*, ten-eleven translocation 2; *lncRNA*, long noncoding RNA; *miRNAs*, microRNAs

## Differences and overlappings among dormant/quiescent cells, drug-tolerant persisters, cancer stem cells, and diapause-like and senescent cancer cells

The existence in tumors of different cellular states characterized by slow/absent proliferation such as dormancy, drug resistance, stem-like, diapause-like states, and even senescence may generate some confusion. Some of these states partially overlap with others and have poorly defined boundaries, and all are dependent on microenvironmental signals. Despite such complexity, we have made an attempt to point out similarities and differences among quiescence, persistence, stemness, diapause-like, and senescence in Table 2. Dormant/quiescent cells (including quiescent cells found in therapy-treated tumors but also in untreated tumors and DTCs present in pre-metastatic sites) are primarily characterized by very slow or absent proliferation. In addition to their slow or non-proliferative state, QCCs are usually characterized by an increased expression of factors implicated in stemness, EMT, stem cell plasticity, and drug resistance [3]. Persister cells (or DTPs) are primarily defined by their drug-resistant state that can originate from genetic mutations, epigenetic programs, or both. DTPs are usually (but not necessarily) slow-growing cells and may be endowed with tumor-repopulating capacity [108]. CSCs are functionally defined by their tumor-repopulating ability, which can originate from genetic and/or epigenetic determinants [109]. CSCs are also characterized by drug resistance and metastatic ability [3]. CSCs can be either quiescent/slow-growing or rapidly proliferating, as observed in CRC [110], while in several other tumors they adopt prevalently a quiescent phenotype. Finally, diapause-like cells are slowly proliferating/quiescent tumor cells isolated by virtue of their drug-resistant state, thus being largely overlapping (if not identical) to DTPs. They show a diapause-like molecular adaptation consisting of a typical signalling pattern (downregulation of cMyc and mTOR activity, autophagy dependence) with no specific genetic alteration [49, 50]. Due to the overlapping and interdependence of quiescent, chemoresistant, and CSCs populations, a better definition should consider these states as highly intertwined metastable processes rather than separated entities. While QCCs, DTPs, CSCs, and diapause-like states are transient stemness-related states, senescence is a stable form of cell cycle arrest that does not imply the activation of stemness programs. Senescence-related arrest occurs prevalently in G1, differently from quiescence, which happens in G0. Previously believed to be a passive and irreversible cellular state, senescence is emerging as a highly dynamic process with an intense crosstalk with the microenvironment [111]. Moreover, under certain circumstances, senescent cells can re-enter the cell cycle or be reprogrammed into iPSCs [112, 113]. In a nutshell, cellular states of slow/absent proliferation are a double-edged sword in cancer therapy, on one side restraining tumor expansion but on the other side allowing tumor resilience.

**Table 2:** Cellular states related to stemness, quiescence, and therapy resistance

	Cellular state in cancer	Cell name	Genetic mutations	Epigenetic alterations	Primary features	Secondary features	Representative refs
1	Quiescence	QCCs DTCs	No	Yes	Slow/absent proliferation (G0 arrest)	Enhanced stemness, EMT, plasticity, drug resistance	[3, 37, 38, 114]
2	Persistence	DTPs	Sometimes	Yes	Drug resistance	Quiescence (most frequently), plasticity	[115, 116]
3	Stemness	CSCs	Sometimes	Yes	Tumor-repopulating capacity, self-renewal	Drug resistance, plasticity, metastatic capacity	[117]
4	Diapause-like	diapause-likeDTPs	No	Yes	Drug resistance	Quiescence	[49, 50]
5	Senescence	Senescent cell	No	Yes	Growth arrest (G1, sometimes G2)	SASP	[111]

Abbreviations: *QCCs*, quiescent cancer cells; *DTCs*, disseminated tumor cells; *EMT*, epithelial-mesenchymal transition; *DTPs*, drug-tolerant persisters; *CSCs*, cancer stem cells; *SASP*, senescence-associated secretory phenotype

## Role of the tumor microenvironment in the acquisition of dormancy

A picture of cancer cell quiescence would not be complete without a mention of the tumor microenvironment (TME) role in the induction and maintenance of dormancy. The interactions between dormant cancer cells and the TME can be roughly divided in three categories that will be briefly discussed below. For a detailed analysis of each category, we refer to excellent reviews on each specific topic. Notably, each category of quiescence-inducing signals is not separated and mutually exclusive with the others. Rather, quiescence-inducing signals from the TME coexist and cooperate with each other, often converging on the same intracellular pathways to maintain the dormant state.

### Paracrine signals involved in cancer quiescence (secreted factors and exosomes)

Cancer cell quiescence crucially depends on soluble factors secreted by TME cells. To cite a few, growth arrest-specific protein 6 (GAS6) and bone morphogenetic protein 7 (BMP7) were shown to induce quiescence in multiple kinds of cancer cells infiltrating the bone marrow [118]. Thrombospondin-1, a glycoprotein secreted by endothelial cells, has been shown to induce quiescence of breast cancer cells within perivascular niches located in lung, bone marrow and brain [71]. Also osteoclast-secreted factors such as growth differentiation factor 10 (GDF10) and TGFβ2 are implicated in inducing tumor cell dormancy [60]. Several extracellular mediators of quiescence converge on inducing a p38^high^/ERK^low^ state, resulting in cell cycle arrest or slowdown [55]. Recently, soluble factors released by macrophages have been reported to induce NR2F1 and dormancy in disseminated breast cancer cells [119]. Exosomes are intraluminal vesicles with an average diameter of 100 nm that contain intracellular components including proteins, microRNAs (miRNAs), and messenger RNAs. Exosomes play a critical role in cancer cell communication and unsurprisingly are emerging as important mediators of chemoresistance, EMT, and dormancy [106, 120, 121]. The effects of miRNAs shuttled within exosomes have been studied particularly in breast cancer, where miR-23b and miR-222/223 have been shown to induce a dormant phenotype in tumor cells [122, 123].

### Juxtacrine signals involved in cancer quiescence (extracellular matrix and cell–cell interactions)

The extracellular matrix (ECM) is a critical component of the TME, being a key regulator of cancer progression and metastasis. Specific ECM proteins have been shown to be implicated in cellular dormancy and reawakening, as has been investigated particularly in breast and lung cancer [121, 124]. Recently, a population of dormant LGR5^+^ p27^+^ colorectal CSCs was reported to be enriched upon chemotherapy and was supported by cell-ECM interactions occurring through COL17A1, a hemidesmosome protein mediating cell adhesion to the basement membrane [25]. An important quiescence-inducing mechanism acts at the ECM level through the modulation of lysyl oxidase (LOX) activity and consequent collagen deposition. Collagen production determines matrix stiffness and regulates the balance between tumor dormancy and proliferation [125]. Cell–cell interactions through receptor-ligand binding, adhesion molecules, and intercellular junctions are also implicated in quiescence induction. CSCs have been reported to interact directly with TME cells through the establishment of gap junctions (GJs), which shuttle cytokines, exosomes, and even mitochondria from one cell to the other. GJs and their content have been specifically implicated in cancer dormancy [126] by shuttling miRNAs and exosomes [127, 128].

### Cancer-immune system interactions involved in quiescence

The host immune system influences all the phases of cancer evolution, restraining or supporting tumor growth. The interactions between immune cells and tumor cells are involved in maintaining the long-term latency of both occult primary and metastatic tumors [124]. Accordingly, states of immune suppression remove the brake imposed by the adaptive immune system to dormant tumor cells, thus promoting metastatic outgrowth. In contrast, the innate immune system and its soluble mediators are implicated in the awakening of dormant tumor cells [129, 130]. Dormant cancer cells are able to escape host antitumor immunity by downregulating the expression of tumor-specific antigens and of major histocompatibility molecules [124]. Moreover, dormant cancer cells may enter immune-privileged niches where they can hide for long periods of time [131]. Finally, some tumors acquire the capacity to cause the death or anergy of immune cells, protecting themselves from immunological clearance [132–135]. A breakthrough in understanding the mechanisms of immune evasion by QCCs has been recently provided by Baldominos et al., showing that QCCs are able to compromise immune cell activity, creating niches where they are protected from T cell-mediated killing [23]. Specifically, QCC niches contained compromised dendritic cells, suppressive fibroblasts, and an increased proportion of terminally exhausted T cells as compared to progenitor T cells [23]. The capacity of QCCs to create an immunosuppressive TME was crucially dependent from a hypoxia-related gene expression signature and possibly from the creation of a glucose-poor and lactate-rich environment as a consequence of tumor metabolism [136].

## Quiescence, stemness, and the effect of anticancer therapies

**Fig. 1:**
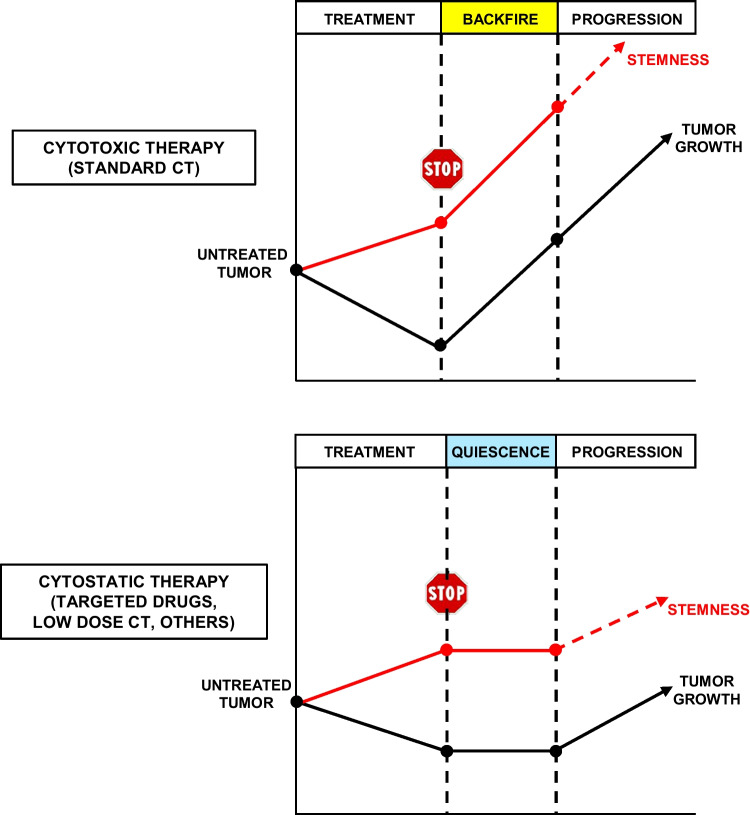
Different effects of cytotoxic therapies and cytostatic therapies on tumor growth and stemness. Simplified representation of the effects of cytotoxic therapies (upper panel) and cytostatic therapies (lower panel) on tumor growth (black line) and stemness (red line) during tumor evolution. CT, chemotherapy

When considering the reciprocal interplay of quiescence, stemness, and therapy resistance, it is important to make a distinction between two main classes of therapies, corresponding to deeply different effects and mechanisms of action. On one side, cytotoxic therapies such as standard chemotherapy achieve, in the best case, tumor regression followed by tumor progression shortly upon treatment interruption (Fig. 1, upper panel). This process is paralleled by drastic perturbations both in the tumor and in the TME, resulting in an abrupt increase of tumor stemness. The tumor-promoting effect of chemotherapy was previously named “treatment backfire” [137], and its mechanisms have been recently discussed in detail [138, 139]. On the other side, cytostatic therapies (including molecularly targeted drugs and low-dose chemotherapeutic regimens) usually achieve a temporary regression/stabilization of responsive tumors, inducing less drastic changes both in the tumor and in the TME. The net result of this process is a state of tumor quiescence lasting for a variable amount of time (Fig. 1, lower panel). Post-treatment quiescence is usually associated with increased stemness traits and has been found to be associated with improved patient survival [34]. In fact, during post-treatment quiescence, tumor cells reside in a state characterized by high stemness and low/absent proliferation, which is clinically more favorable than the aggressive progression phase. In a nonlinear dynamics frame, this observation is in line with the fact that dormancy, corresponding to an invariant state potentially lasting for very long time, is supported by a configuration more similar to the healthy attractor state (which is much more probable with respect to cancer) as compared to the aggressive/destructive cancer attractor. Both cytostatic and cytotoxic treatments end up with a phase of tumor progression characterized by the uncontrolled expansion of therapy resistant cells. In this phase the concept of stemness is lost (dashed red line in Fig. 1) as the boundaries between stem cells and non-stem cells become unclear and most cells become endowed with tumor-repopulating capacity [109]. Importantly, tumor progression that follows cytostatic therapies is less aggressive as compared to that occurring upon standard chemotherapy, often allowing the sequential administration of multiple targeted treatments. However, although in a more gradual fashion, repeated rounds of cytostatic treatments will still increase tumor aggressiveness, ultimately resulting in unrestrained tumor expansion. The different mechanisms of action of cytotoxic versus cytostatic therapies have been experimentally demonstrated in tumor models and further confirmed by clinical observations. Perhaps the most compelling evidence on this topic has been provided by Chan et al. who showed that standard chemotherapy promotes CSC expansion through the activation of cancer-associated fibroblasts (CAFs), thus leading to post-treatment paradoxical tumor growth [140]. By contrast, low-dose chemotherapy achieved tumor stabilization without inducing CAF pro-tumorigenic signalling, thus enhancing treatment response and the survival of mice carrying breast and pancreatic tumor xenografts [140]. Additional support to this model has been recently provided by the observation that in lung cancer primary cells and xenografts, chemotherapy induced the expression of the stem cell marker CRIPTO resulting in aggressive tumor cell expansion [141]. In line with this observation, an accelerated tumor regrowth has been reported in lung cancer patients upon chemotherapy treatment [142, 143]. A new mechanism of tumor evolution triggered by chemotherapy has been recently described by Musella and coworkers who showed that drug-induced immunogenic cell death stimulates interferon-I (IFN-I) production by breast tumors. In turn, IFN-I reprograms cancer cells toward a more aggressive stem-like phenotype by upregulating KDM1B, acting as an engine of cancer stemness and reprogramming [96]. In contrast, several cytostatic treatments such as clinically approved targeted drugs have been shown to achieve tumor regression or stabilization without inducing a significant stemness increase and subsequent aggressive tumor regrowth. To cite a few examples, the anti-epidermal growth factor receptor (EGFR) antibody cetuximab was shown to block the growth of KRAS wild-type colorectal tumor xenografts without increasing CSCs content [144]. The retinoic acid derivative nanofenretinide inhibited the growth of lung and colorectal xenografts, at the same time avoiding stem cell enrichment and tumor backfire [145]. As the tumor-promoting effects of cancer therapies become more and more evident, strategies aimed at counteracting therapy-induced tumor stemness and backfiring should be investigated in clinical settings.

## The apparent paradox of quiescent therapy-resistant cells in untreated tumors

**Fig. 2:**
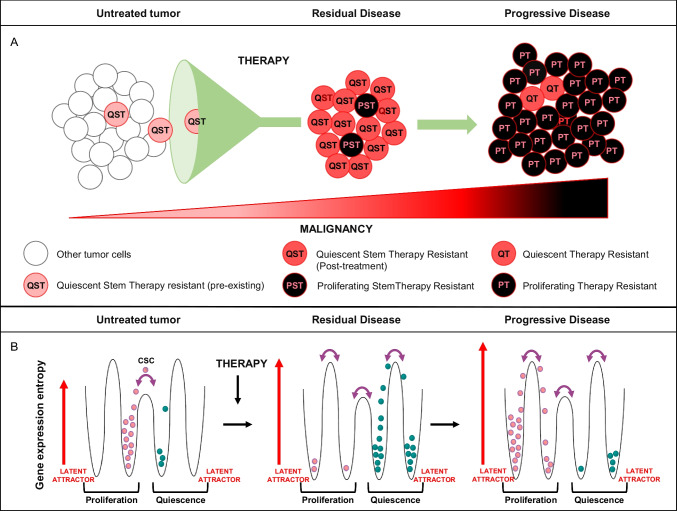
Evolution and proliferative state of therapy resistant cancer cells during tumorprogression. **A** Evolution of therapy resistant cells during the transition from untreated tumor (left) to residual disease (center) to progressive disease (right). Increasing tumor cell malignancy is indicated with progressively dark shades of red (lower triangle). QST, quiescent stem therapy resistant cell; PST, proliferating stem therapy-resistant cell; QT, quiescent therapy-resistant cell; PT, proliferating therapy-resistant cell. In advanced tumors (progressive disease), the majority of cells is characterized by therapy resistance and tumor-initiating potential, and stemness is not clearly definable. **B** Gene expression entropy increases during tumor evolution, allowing tumor cells to occupy previously latent attractors corresponding to new quiescent and proliferative states. Left: in untreated tumors, a cancer stem cell (CSC, on the top of the hill) can occupy attractors corresponding to either proliferative or quiescent phenotypes but does not have enough potential energy to occupy latent attractors. Center: anticancer therapy increases gene expression entropy, allowing CSCs to reach previously unexplored attractors and to adopt new phenotypes. During residual disease, cancer cells occupy prevalently the quiescent attractors, although few cells are also found in a proliferative state. Left: during progressive disease, most cells are found in a new attractor corresponding to an unrestrained proliferating state

Despite the fact that therapy resistance is a condition typically emerging upon anticancer treatments, a population of drug-resistant cells present before any kind of treatment has been identified in many tumors [6–8, 10–14, 16, 18, 19, 98, 146, 147]. Importantly, such population of preexisting slow-cycling drug-resistant stem cells has been recently demonstrated to be the origin of DTPs [26, 148]. The counterintuitive presence of therapy resistant cells in therapy-naïve tumors was shown to be the result of stochastic state transitions inducing a high transient expression of resistance factors [9, 15, 148]. However, interactions with microenvironmental elements and with the immune system play a key role in the determination of preexisting chemoresistant QCCs, as indicated by previous evidences [24, 115] and by recent studies discussed below. An important implication of the strict interdependence between preexisting QCCs and the TME is that in an unperturbed microenvironment, QCCs would be a relatively stable population (Fig. 2A, left). In line with this hypothesis, QCCs have been isolated from untreated colorectal tumors as a long-standing population of label-retaining cells presents after several weeks of xenograft growth [12]. Perturbations of the TME induced by chemotherapy, radiotherapy, or targeted drugs would then disrupt QCCs stability promoting a transition of surviving tumor cells toward a more malignant state driven by both selective and instructive forces [36]. On one side, cells that go through the bottleneck of cancer treatment can be traced back to preexisting QCCs (Fig. 2A, center). On the other side, the near-death experience of cytotoxic treatments produces global changes in gene regulatory networks (GRNs) of surviving cells, resulting in the expression of stress response genes as well as repair genes and stemness-related genes [2, 149]. While the majority of cells composing residual tumors after cytostatic treatments is quiescent, some persister cells have been shown to maintain proliferative capacity in the presence of drugs [150] (Fig. 2A, center). The subsequent evolution of treated tumors consists of the acquisition of a more aggressive phenotype (Fig. 2A, right). In this stage, the majority of cells actively repopulate the tumor and transmit both genetic and non-genetic traits of drug resistance to their progeny [36].

## Origin and dynamics of drug-resistant quiescent cancer cells

What happens to cells that survive anticancer treatments? Non-killed cells have gone through a massive stress that affects their whole transcriptional landscape. Consequently, they are pushed into yet unexplored states characterized by the expression of stress response genes and stemness- and multipotency-related genes [2]. Like any physical system, even cell trajectories in gene expression phase space follow the principle of energy minimization, getting entrapped into minimal energy states called “attractors” that correspond to a given phenotype. A simple metaphor could be a marble in a cup that reaches the bottom of the cup and tends to go back to this minimum energy state even if we (gently) continue to move the cup in our hands. The increase in the amplitude of fluctuations (entropy) of gene expression values makes it possible to escape the minimal energy attractor, analogously to what happen if we increase the motion of the cup. Entropy increase occurs upon perturbations imposed by cancer therapy, allowing the cells to explore the “world outside the cup.” Cancer cells then get entrapped into different attractor states governed by different GRNs. Figure 2B gives a sketchy explanation of this phenomenon. In the untreated tumor, the trajectories of cell populations progress along a hierarchical path going from an apex state (stem cells) endowed with elevated potential energy toward stable equilibrium states (Fig. 2B, left). As aptly stated in [33], while the path of normal stem cells must obey to stringent constraints that drive its trajectory toward a fully differentiated state, CSCs primed by therapy are endowed with an elevated gene expression entropy and consequently experience much wider fluctuations. This entropy increase implies the possibility to explore a wider area of the phase space and confers to these cells the opportunity to reach normally unexpressed (latent) attractors correspondent to more aggressive phenotypes (Fig. 2B, center). These latent attractors display “atavistic” features, being characterized by the increased expression of genes relative to unicellular condition [151, 152] and consequently free of the constrains imposed by multi-cellularity to uncontrolled growth (Fig. 2B, right) [153]. The biologic origin of preexisting drug-resistant cells was investigated in recent studies that provided important insights on how cell intrinsic and cell extrinsic factors cooperate to shape this population. In breast cancer, preexisting QCCs resistant to HER2 TKIs were recently identified through a lentiviral barcoding strategy and showed to be the cells of origin of drug-tolerant persisters evoked by targeted treatment [148]. Quiescent tumor cells expressing stemness and chemoresistance genes were also isolated from chemo-naïve TNBC as cells able to survive T cell-mediated killing [23]. The latter observation suggests that preexisting QCCs may arise early during tumor evolution as a population able to evade immune surveillance and subsequently find a selective advantage during cancer treatment. Finally, several studies agree on the fact that preexisting QCCs are the origin of drug DTPs arising upon anticancer treatments. At the same time, other studies suggest that persister phenotypes arise as a consequence of plasticity induced by drug treatment (reviewed in [115]). The existence of both pre-treatment and treatment-induced persister cells is not mutually exclusive. In fact, QCCs in untreated tumors may survive and further evolve during treatment, thus acquiring *de novo* malignant traits ultimately responsible for tumor relapse.

## Role of cancer therapy in tumor evolution: cell intrinsic mechanisms

Both conventional and targeted cancer therapies have been shown to be followed by the emergence of therapy resistant tumors. Recurrent tumors are qualitatively different from untreated tumors, as they contain more aggressive cells expressing factors involved in inflammation and immunosuppression [34]. Emerging bodies of evidence indicate that the backfiring of chemotherapeutic treatments is the expression of protective and regenerative responses jointly orchestrated by tumor cells and by the TME, and not simply the passive selection of fitter preexisting cells [36]. Intrinsic changes in tumor cells induced by cancer therapies have been shown to be the sum of genetic and non-genetic events [154]. An important advancement in understanding genetic changes occurring upon cancer therapy has been recently provided by Lagomarsino and coworkers who showed that targeted drugs induced an up to 50-fold increase in the mutation rate of surviving cells [155], in line with an active role of therapy in the induction of a more aggressive cancer phenotype. At the same time, non-genetic effects of therapy on tumor cells include activation of stress-induced pathways, metabolic reprogramming, and epigenetic modifications, and have been reviewed elsewhere [3]. Genetic and non-genetic models of treatment-associated progression have been recently proposed to synergize in driving post-treatment tumor aggressiveness [4]. An interesting insight on the cell-intrinsic mechanisms linking anticancer treatments and tumor stemness has been recently provided by Vasquez et al. by analyzing intestinal tumors of patients who received preoperative chemotherapy plus targeted therapy. Patients with a higher phenotypic plasticity of the CSCs compartment showed a poorer response to therapy, suggesting that stem cells’ adaptive capacity is tightly related with clinical response to treatment [156].

## Role of cancer therapy in tumor evolution: microenvironment-mediated mechanisms

**Fig. 3:**
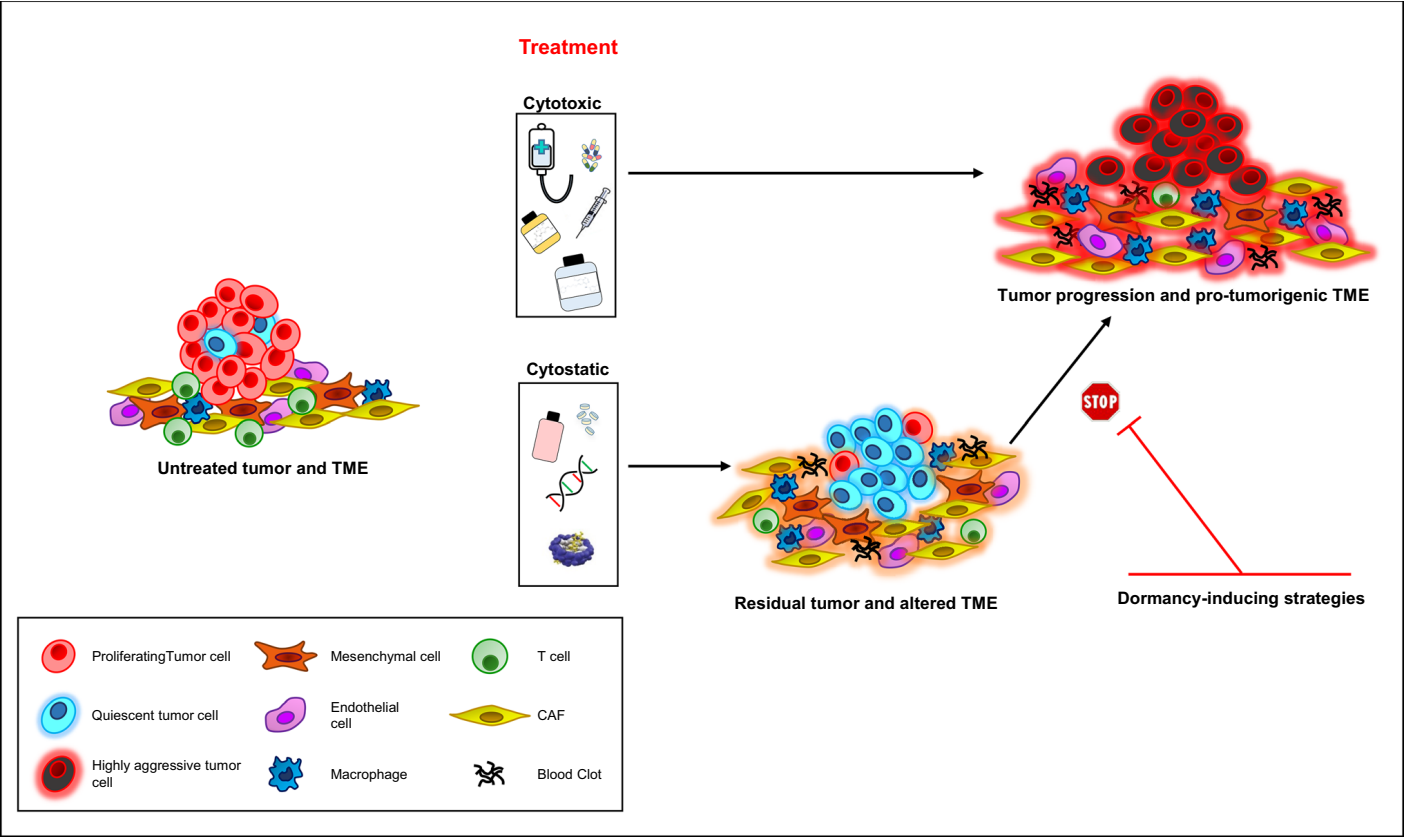
Cumulative effects of cytotoxic versus cytostatic therapies on tumor cells and on the tumor microenvironment (TME). Cytotoxic therapies such as standard chemotherapy induce pro-tumorigenic changes in both cancer cells and microenvironmental cells, resulting in rapid tumorprogression. Differently, cytostatic therapies (including targeted therapies and low-dose chemotherapy) induce TME alterations that are compatible with a quiescent state. Dormancy-inducing strategies acting on tumor cells and on the TME can stabilize the quiescent state and prevent degeneration toward progressive disease

Besides tumor intrinsic changes responsible for drug resistance, an increasing number of studies indicated that the TME plays a crucial role in post-therapy tumor recurrence. Collateral effects of treatment on host cells, collectively grouped under the expression “host response to cancer therapies,” have been demonstrated to promote tumor aggressiveness and metastasization [138, 157]. All the cell types present in the TME including stromal, immune, and endothelial cells, have been shown to be affected by cytotoxic treatments, responding with both damage and establishment of pro-tumorigenic phenotypes [157]. Therapy-induced changes in the TME are qualitatively and quantitatively different in the case of either cytotoxic or cytostatic therapies (Fig. 3). Available bodies of evidence suggest that inflammation is prevalent in the case of standard chemotherapy, while immunosuppression and TME remodelling prevail in the case of targeted therapy. Accordingly, tumor-associated stromal cells were previously shown by Sun et al. to change their spectrum of cytokine production in response to chemotherapy, promoting the survival of cancer cells through WNT16B secretion [158]. Recently, Nicolas et al. revealed a key role of inflammatory CAFs in dictating chemoradiotherapy resistance in rectal cancer. Irradiation had a double effect on CAFs, which were polarized toward an inflammatory phenotype and underwent senescence, resulting in therapy resistance and disease progression [159]. Lately, inflammatory CAFs have been found enriched also in chemoresistant samples of pancreatic cancer [160]. In this study, inflammatory CAFs expressing stem cell markers were a small population in untreated tumors but they triplicated upon treatment [160], suggesting that chemotherapy promotes stemness not only in tumor cells but also in the surrounding stroma. Immune cells present in the TME are also profoundly affected by anticancer treatments. Anticancer therapies have been previously shown to impair immune system activities, increasing the immunosuppressive effects of tumor cells and reshaping immune cell infiltration, leading to tumor-immune escape [161]. The evolution of the immune TME during targeted treatment was recently observed in lung cancer patients where residual disease and progressive disease showed inverted proportions of T cells and macrophages [34]. Macrophages present in post-treatment progressive disease expressed pro-inflammatory cytokines and the metabolic enzyme indoleamine 2,3-dioxygenase 1 (IDO1) that is involved in the generation of an immunosuppressive environment [34, 162]. Notably, chemotherapy has been demonstrated to promote not only chemoresistance but also metastasization by inducing both local and systemic pro-tumorigenic and pro-metastatic factors, as has been addressed by excellent reviews [138, 163]. In this regard, recent insights provided by Haj-Shomaly et al. showed that chemotherapy induces a prometastatic remodelling of the pulmonary ECM mediated by CD8^+^ T cells. Paclitaxel promoted ECM remodelling through LOX upregulation in T cells, whereas LOX inhibitors suppressed the pro-metastatic effects of chemotherapy [164]. Finally, cancer cell intrinsic and cell extrinsic changes induced by targeted KRAS G12C inhibitors have been recently analyzed in tumor autopsies of a lung cancer patient, providing a real-life picture of the pro-tumorigenic effects of treatment on both tumor cells and the TME. Treatment-induced changes in tumor cells included bypassing KRAS inhibition, metabolic reprogramming, and EMT, while changes in the TME consisted of increased coagulation, angiogenesis, and immune suppression [165].

## Shifting the balance from aggressive to nonaggressive tumor phenotypes: quiescence as a therapeutic opportunity

Precision oncology has been defined as the concept of cancer treatment strategies that are based on the distinct molecular characteristics of a tumor [166]. An implication of this concept is that the combined or sequential use of targeted drugs would maximize the resultant antitumor effect. In fact, a more extensive alteration of the edges of an interaction network prevents the activation of bypass and alternative signalling pathways [167]. This is true for “flow” networks (i.e., for metabolic networks in which the nodes are metabolites and edge the chemical reactions between them) and is the basis of the genetic concept of synthetic lethality [168]. However, the case of gene co-expression networks governing cancer evolution is drastically different. In fact, these networks are not “flow” networks but “influence” networks where the activity of one node influences the activity of other nodes connected to it. This ends up into a global state of the network node activation consistent with their mutual constraints. Moreover, the actual gene co-expression network (i.e., the empirical correlation between different gene expressions) widely varies in different cell kinds and states: accordingly, quiescent and proliferating stem cells have different gene co-expression networks conserved among different tumors [44, 46]. Last but not least, the effective phenotype of a co-expression network is not static but dynamic and has to do with the ability of the same network wiring structure to support different stable phenotypes (attractors) in the multidimensional gene expression phase space [5]. This implies that even if multi-target approaches can provide a survival benefit as compared to single targeted drugs (as in the case of pan-HER inhibition in CRC xenografts, [169]), we need a different strategy taking into consideration both the complexity and resilience of cancer gene co-expression networks and the dynamic nature of cancer phenotypes. One possibility is to focus therapeutic strategies in the direction of stabilizing non-aggressive phenotypes such as quiescence, shifting the balance from tumor progression to chronic tumor dormancy (Fig. 3). Strategies aimed at prolonging the dormancy of residual tumor cells such as hormone therapies or CDK4/6 inhibitors are used in clinical settings as a mainstay treatment for breast and prostate cancer [170, 171]. Experimental strategies aimed at increasing the expression of dormancy-related factors such as p38 and NR2F1 have also been shown to induce cancer dormancy and prevent metastatic outgrowth [53, 65]. Despite these promising bodies of evidence, the clinical application of dormancy-inducing strategies has a difficult time in finding a broad clinical application [31]. A major concern of dormancy-inducing strategies consists of unwanted side effects that limit long-term utilization and patient compliance. For this purpose, the use of fenretinide derivatives [145, 172] or all-trans retinoic acid (ATRA)/5-azacytidine combination (the latter being currently evaluated in clinical trial NCT03572387) may be a feasible and low-toxic strategy. A promising field of intervention for therapeutic strategies aimed at preventing tumor recurrence consists in modulating tumor metabolism. Specifically, the metabolic pathways involved in tumor cell dormancy have been reported to exploit oxidative phosphorylation, reactive oxygen species (ROS) scavenging, and autophagy to ensure energy supply [173]. To this end, multipurpose drugs acting on cell metabolism such as metformin and peroxisome proliferator-activated receptor gamma (PPARγ) agonists have been shown to inhibit tumor growth and inflammation [174, 175]. In particular, PPARγ agonists were shown to be effective in combination with a broad variety of systemic therapies [176]. Metabolic modulators, low-dose chemotherapy, epigenetic agents, retinoic acid derivatives, and a number of repurposed drugs have been all considered in the frame of *anakoinosis*, a therapeutic strategy based on tumor communicative reprogramming [177, 178]. Finally, senescence modulation is attracting increasing interest in cancer treatment [179]. Senescence-inducing therapies (such as low-dose chemotherapy and several targeted agents) may restrain tumor growth by inducing stable cell cycle arrest in tumor cells. However, senescent cells secrete an array of factors that, in the long term, promote tumor growth [179]. Therefore, senolytic therapies may be sequentially employed after senescence-inducing treatments in order to eliminate senescent cells, thus attenuating senescence-induced inflammation and preventing the reawakening of dormant persisters [180].

## Dormancy-inducing strategies acting on the tumor microenvironment

A number of therapeutic strategies acting on TME stromal or immune components have been recently reported to counteract chemotherapy-induced inflammation and/or to promote tumor dormancy. Compounds targeting matrix metalloproteinase, hedgehog signalling, and TGFβ signalling have been shown to inhibit the tumor-promoting effects of CAFs [181]. Interestingly, ATRA has been shown to induce the quiescence of stromal cells in pancreatic adenocarcinoma resulting in decreased tumor proliferation and stemness [182]. Thus, retinoic acid derivatives may play a double role by inducing antiproliferative and antimetabolic effects both on tumor cells and on the surrounding stroma given their pleiotropic mechanism of action [145, 172, 182]. Targeting microenvironmental acidity with proton pump inhibitors has also shown to effectively restrain tumor cell growth [183]. Recent advances in targeting the pro-tumorigenic effects of tumor stroma include the use of IL1α blocking antibodies. In fact, blocking IL1α signalling has been shown to revert CAF inflammatory phenotype, thus reducing tumor growth and chemoradiotherapy resistance in rectal cancer [159]. In addition to CAFs-targeted approaches, immune targeted strategies have been shown to inhibit tumor cell reawakening and prolong cancer dormancy. Interleukin-15 immunotherapy has been shown to ensure a pool of natural killer (NK) cells supporting the dormancy of breast cancer hepatic metastases [184]. Blocking integrin β1 activation by neutrophil extracellular traps prevented the awakening of breast cancer lung metastases [130]. The anti-inflammary autacoids resolvins have been shown to counteract the release of pro-tumorigenic cytokines by macrophages stimulated by antitumor therapies, thus counteracting therapy backfire and suppressing tumor growth [185]. A comprehensive strategy to reshape both systemic and intratumor immunity consists in fasting-mimicking diet (FMD), which has been reported to induce important metabolic changes and to activate antitumor immune programs in patients with different tumors and treated with different antitumor therapies [186]. Finally, lifestyle-related factors such as diet and exercise have been shown to reinforce the immune system, prevent inflammation, re-equilibrate the gut microbiota, and modulate hormone levels, thus generating environments that promote tumor dormancy by acting at both local and systemic levels [3, 35].

## Conclusions

Our understanding of tumor quiescence and stemness has advanced considerably over recent years, yet it has still to be translated in the clinical setting. While the mechanisms responsible for the emergence of therapy resistance are being progressively elucidated, the intertwining of therapy resistance with quiescence and stemness is also becoming increasingly clear. Here, we discussed recent insights into the field of tumor quiescence that intersect with the related fields of cancer stemness and therapy resistance. We foresee that while common factors and gene expression programs continue to emerge, these fields will be more and more considered as inseparable. Future efforts to identify new mechanisms, biomarkers, and vulnerabilities of quiescent therapy-resistant stem cells will likely open new avenues to prevent or delay tumor recurrence. These future developments will require a deeply modified point of view with respect to the “magic bullet” paradigm toward a systemic approach fostered by a dynamical systemic view of cancer development trajectories in time encompassing cancer cell heterogeneity and microenvironment issues.
